# Morphological description of the glochidia of *Buldowskia suifunica* (Bivalvia: Unionidae)

**DOI:** 10.1038/s41598-023-46894-3

**Published:** 2023-11-13

**Authors:** Elena M. Sayenko, Viktoria E. Nikischenko, Vyacheclav A. Dyachuk

**Affiliations:** 1 Federal Scientific Center of the East Asia Terrestrial Biodiversity, Far Eastern Branch, Russian Academy of Sciences, Vladivostok, Russia; 2 A.V. Zhirmunsky National Scientific Center of Marine Biology, Far Eastern Branch, Russian Academy of Sciences, Vladivostok, Russia

**Keywords:** Developmental biology, Zoology

## Abstract

Freshwater mussels of the genus *Buldowskia* (Bivalvia, Unionidae) are distributed from the Amur River basin in Russia and China southward to the Korean Peninsula and some Japanese islands. This work is an integrative morphological study of *Buldowskia suifunica* glochidia from locations in the Primorsky Territory, the Russian Far East. Glochidia of *B. suifunica*, taken from the same gill have asynchronous development. The external and internal morphology of its shell has been characterized. The morphology of its sensory system, within three stages of larval development (immature, intermediate and mature glochidia), consists of hair cells as well as nonhair cells. Their muscle system is composed of massive adductor and minor muscle bundles. The FMRFamid-ergic nervous system turned out to be a complex system includes basal cells (neurons), their neurites and anterior neurons. FMRFamide and tubulin was found in all neurons. Glochidia of *B. suifunica* have only four 5-HT-lir neurons. We concluded that *B. suifunica* glochidial nervous system differs from those of the larval systems of planktotrophic marine mollusks.

## Introduction

The life cycle of most freshwater bivalves includes a special larva (lasidia or haustoria in Etherioidea and glochidia in Unionoidea), which parasitizes fish^1–6^ for some time prior to metamorphosis. Only very few Unionidae species are known to successfully infect amphibians with glochidia, namely, tadpoles and aquatic salamanders^7–10^. Glochidia may also attach to invertebrate hosts such as glossiphonid leeches^7^ or decapod crustaceans^11^ without metamorphosing.

As an obligatory parasitic stage, glochidium represents a specialized variant of the planktonic larva of marine mollusks—–a veliger—, so a temporal attachment to a suitable host is needed for nutrition and dispersal of the larvae^1–7,10,12^.

The general structural plan of unionid glochidia is the same as in adult bivalves. They have two valves and a dorsal hinge, although the shape and details of glochidial morphology may vary among the taxa. Glochidia larvae can be subrotund, subtriangular, semioval, subelliptical, range from 60 to 450 µm, hooked or hookless^6,13,14^. The glochidial shell consists of two layers, the thick porous inner layer and the thin outer layer, which forms a special external microsculpture and covers the outer ends of the pores^15^. With high magnification, ridges and depressions, which appear to be organized in a regular manner, can be observed on the external shell surface^13,16,17^.

The inner surface of the glochidial valves is covered with the larval mantle bearing bundles of sensory hairs along the mantle edge^6,18^. Unlike adult unionids, the glochidium has only one adductor muscle that degenerates during metamorphosis and is replaced by a pair of adductor muscles in a juvenile mussel^6,18^. Gills, intestines and feet are rudiment in the glochidium^6,18^. As a result of metamorphosis, internal organs such as the mouth, anus and digestive tract appear in juveniles but are absent in the glochidium^6,18^. To attach successfully to the host fish, glochidia of some taxa have a long sticky larval thread^6,13^. There are some variations in the form, position, presence or absence of the larval thread among unionid species^6^.

Some of the abovementioned characters of glochidia allow us to differentiate bivalve taxa, such as unionin and anodontin freshwater mussel^6,13,14,16,17^.

The anodontin genus *Buldowskia* was described in 1973 by Moskvicheva during the analysis of freshwater bivalve species from the Amur River basin and water bodies of Primorye (the Russian Far East). Subsequently, eight species of *Buldowskia* were identified based on differences in shell shape^19–22^.

Based on genetic data, the presence of five *Buldowskia* species was supported, one of which, namely, *Buldowskia shadini* (Moskvicheva, 1973), was moved from the closely related genus *Anemina*^23^. It is noted that *B. shadini* has a wide disjunct distribution throughout the Amur River basin in Russia and China, the Buir Lake basin in Mongolia and the major river basins of South Korea (data from North Korea are lacking). The others species are: *B. suifunica* (Lindholm, 1925) inhabits the Razdolnaya River basin and the coastal rivers southwest of Vladivostok (southern Primorsky Territory in Russia) to North Korea; *B. flavotincta* (Martens, 1905) is endemic to three river basins (Hangang, Geumgang, and Nakdonggang) in South Korea; and two other species *B. iwakawai* (Suzuki, 1939) and *B. kamiyai* Sano, Hattori & Kondo, 2020 are native to Japan^23,24^. The phylogenetic relationships among species in the genus are still not well resolved^23,24^.

Glochidia of *Buldowskia*, including taxa first mentioned as members of the *Anemina* genus and later synonymized with *Buldowskia*, were studied using both light and scanning electron microscopes. The first brief descriptions of *B. shadini* glochidia were given by Zhadin^25^ and Inaba^26^. Later, a number of authors continued to study glochidia morphology and the reproductive cycle of this group of mussel using light microscopy^14,21,27–30^ and scanning electron microscopy^31–35^.

During many years, investigations of unionid glochidia (including anodontins, in particular *Buldowskia*) have mainly focused on the morphology of glochidial shells. As a result, little is generally known about embryonic development, specifically the anatomy of glochidia during metamorphosis into juveniles. It was shown that their metamorphosis encompasses a few distinct stages, with some variations between investigated taxa. The first involves degeneration of the single larval adductor muscle and formation of the characteristic mushroom body^18,36,37^. The final stage involves the formation of the major anatomical structures and organ systems in juveniles^18,38,39^.

Morphological investigations of Unionidae glochidia, including their specialized neurostructures, need to be further studied for comparison with the anodontin family members.

Here, we present new data on the structures and morphology of glochidial shells using light and scanning electron microscopy as well as the immunostaining protocol developed by us in combination with confocal microscopy for the detection of the sensory, muscular and nervous systems in larvae.

## Results

### General morphology of glochidia

**Figure 1:**
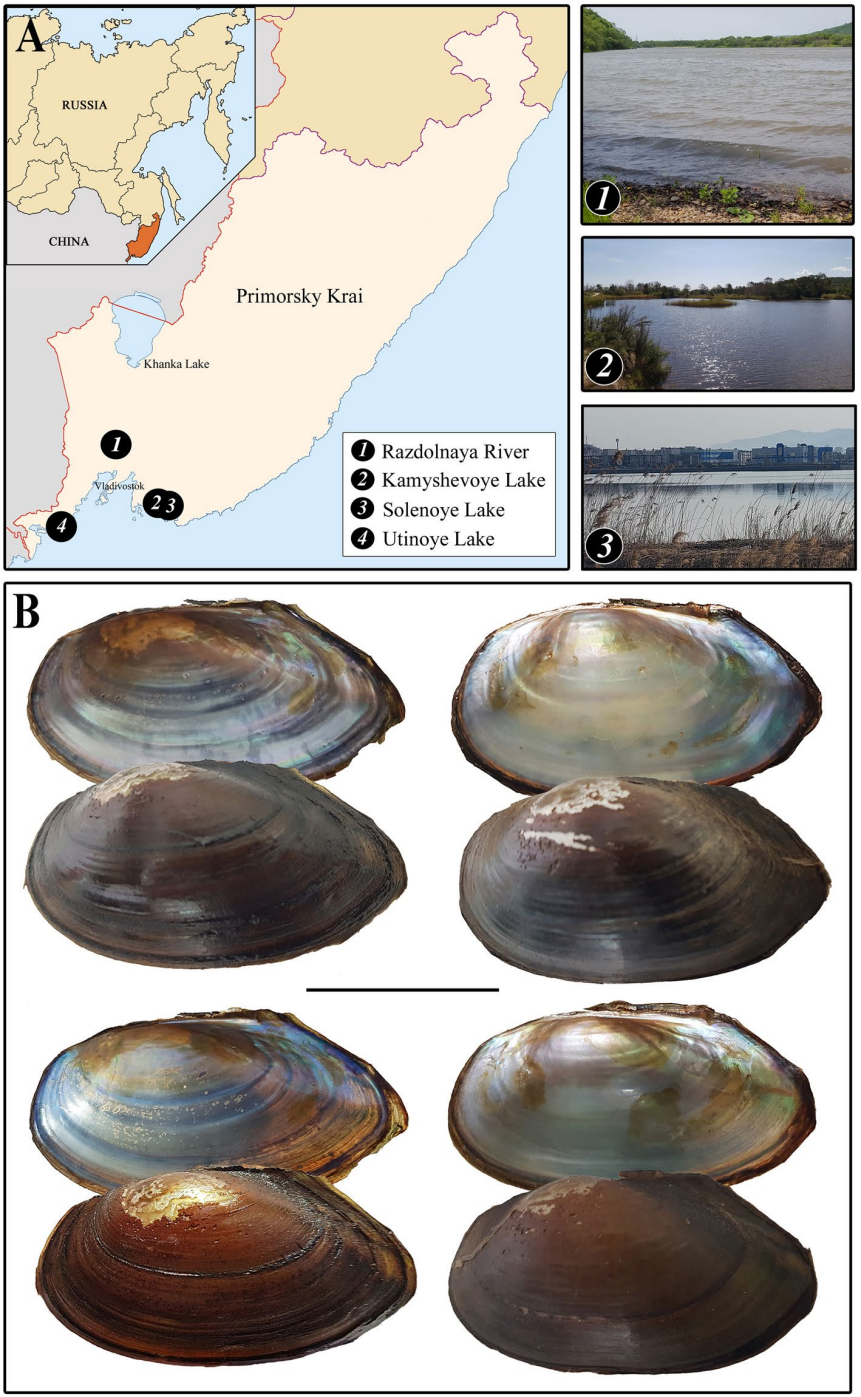
(**A**) Map with selected views of sampling localities. (**B**) Adult shells of *Buldowskia suifunica* whose glochidia were used in the investigation (Kamyshevoye Lake). Scale bar 5 cm. Photo by E. Sayenko (**1**, **2**) and O. Nakonechnaya (**3**). The figure with a map was created using Adobe Photoshop Elements 9 software; the topographic base of the map was taken from free sourse (https://d-map.com).

**Figure 2:**
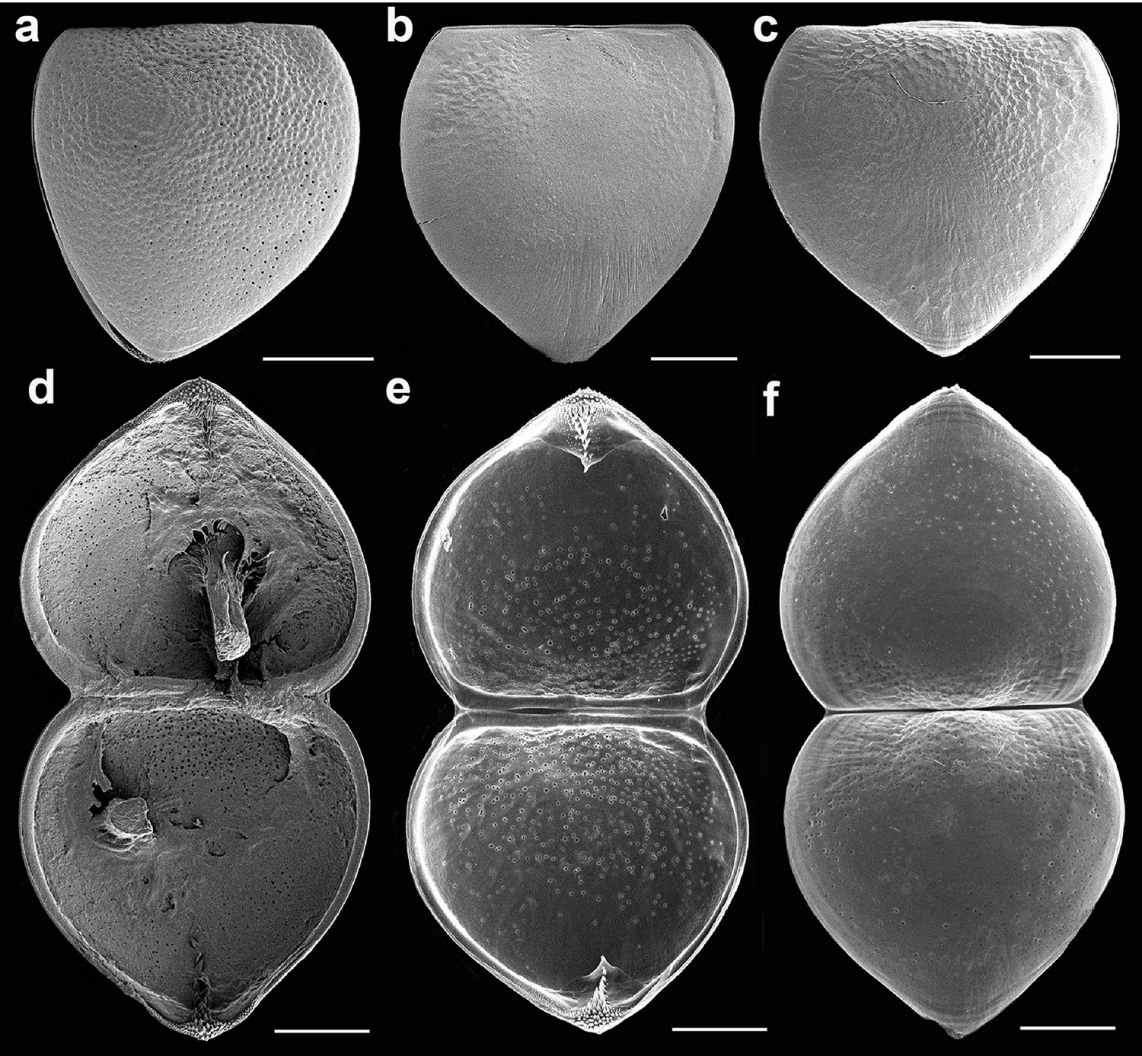
Mature glochidia of *Buldowskia suifunica*, closed (**a**–**c**) and open (**d**–**f**) shells. (**a**) Solenoye Lake, 22/VI/2017; (**b**) Kamyshevoe Lake, 16/IX/2017; (**c**) Kamyshevoe Lake, 21/IX/2020; (**d**) Utinoye Lake, 11/XI/2005, interior view, the remains of a ruptured adductor are visible inside both valves; (**e**, **f**) Kamyshevoe Lake, 17/IV/2022, interior and exterior views. SEM. Scale bars 100 µm.

**Figure 3:**
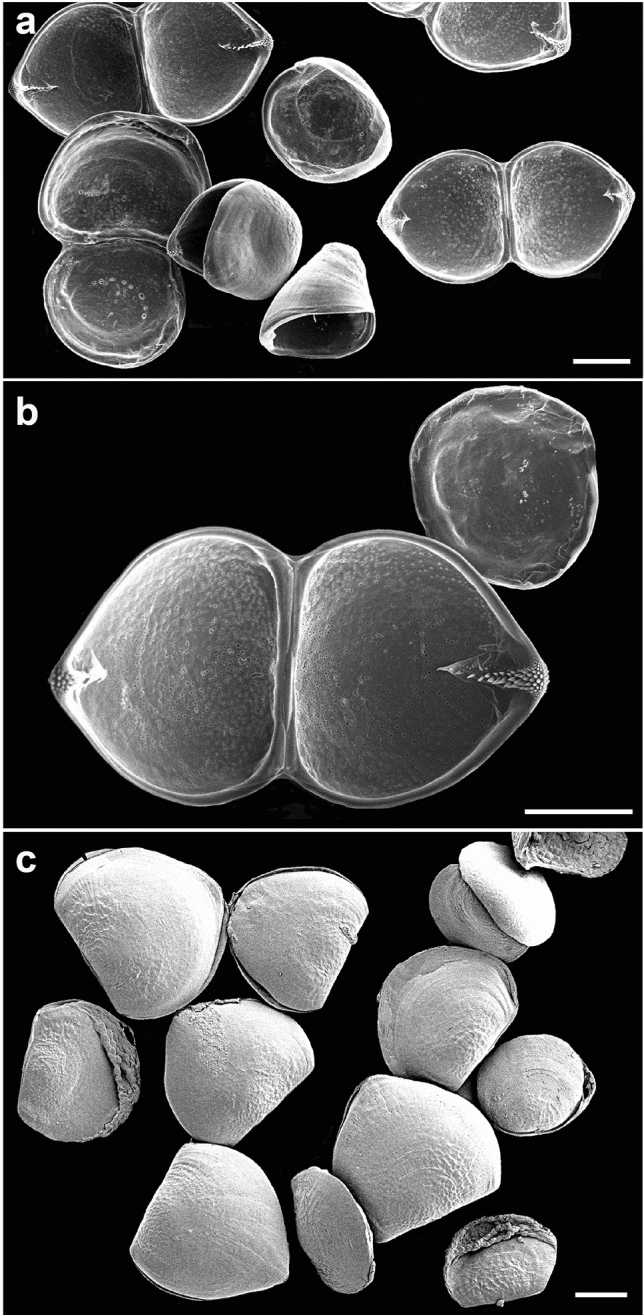
Glochidia from a single gill demibranch of *Buldowskia suifunica* with various degrees of maturity. (**a**, **b**) Kamyshevoe Lake, 17/IV/2022; (**c**) Kamyshevoe Lake, 21/IX/2020. SEM. Scale bars 150 µm.

**Figure 4:**
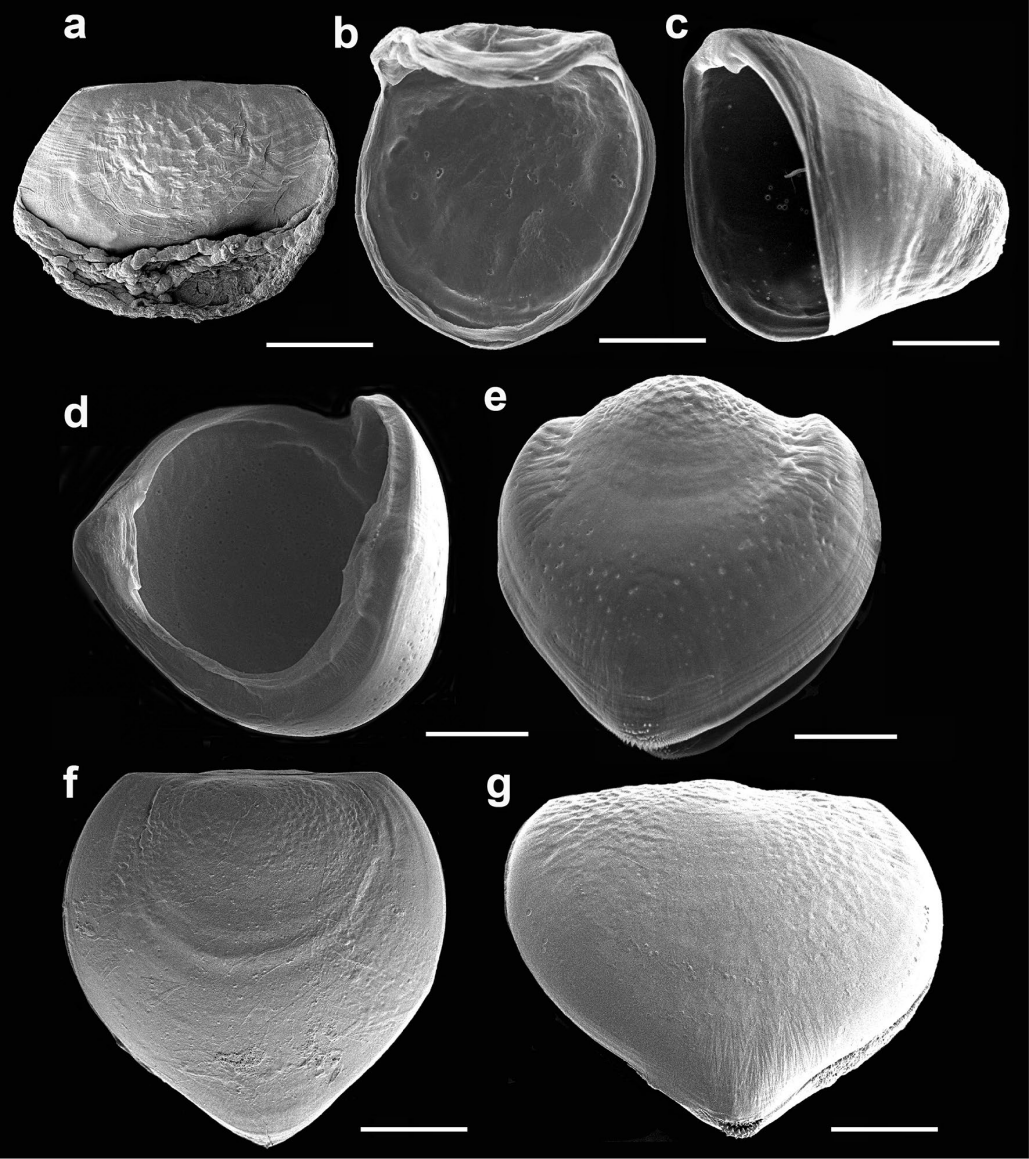
Glochidia at different stages of development. (**a**) Immature glochidium with the cap-like shell, soft tissues are visible between the slightly ajar valves (Kamyshevoe Lake, 21/IX/2020); (**b**) immature subrotund shell (Kamyshevoe Lake, 17/IV/2022); (**c**) immature shell, the tip of the larval thread is visible inside (Kamyshevoe Lake, 17/IV/2022); (**d**) ajar immature shell, lateral view (Kamyshevoe Lake, 17/IV/2022); (**e**) immature shell with protruded dorsal parts of the valves (Kamyshevoe Lake, 17/IV/2022); (**f**) subtriangulate shell with the growth lines (Utinoye Lake, 11/XI/2005); (**g**) mature subtriangulate shell, view from the ventral angle of the shell, macrospines of the hooks are visible (Kamyshevoe Lake, 21/IX/2020). SEM. Scale bars 100 µm.

Specimens of *Buldowskia suifunica* were collected from three localities in Primorye, the Russian Far East (Table 1; Fig. 1). Glochidial shells of *B. suifunica* are typically anodontin hooked shells and subtriangular in shape with the ventral angle protruding dorsally (Fig. 2). Mature glochidia are large, up to 395 µm in height and 407 µm in length, and elongated longitudinally, so the length of the glochidial shell is always greater than its height. Glochidia from a single gill demibranch vary in size considerably, up to 45 µm, because of the simultaneous presence of mature and developing immature larvae (Figs. 3, 4).

**Table 1:** Data on glochidia samples of *Buldowskia sujfunica* used for the light (LM), scanning electron (SEM) and confocal (CM) microscopy investigations.

Locality, date, collectors	number of samples (= adult specimens)	method of investigation
Primorye, *Razdolnaya River* basin, 13/XI/1993; E.M. Sayenko	1	LM
Primorye, Troitsa Bay, *Utinoye Lake*, 11/XI/2005; A.Yu. Semenchenko	1	LM, SEM
Primorye, Nakhodka Bay, *Solenoye Lake*, 22/VI/2017; E.V. Kolpakov	1	LM, SEM
Primorye, Rifovaya Bay, *Kamyshevoye Lake*, 16/IX/2017; E.M. Sayenko, I.A. Rodionov	2	LM, SEM, CM
Primorye, Rifovaya Bay, *Kamyshevoye Lake*, 21/IX/2020; E.M. Sayenko, I.A. Rodionov	5	LM, SEM, CM
Primorye, Rifovaya Bay, *Kamyshevoye Lake*, 17/IV/2022; E.M. Sayenko, I.A. Rodionov	5	LM, SEM, CM

**Figure 5:**
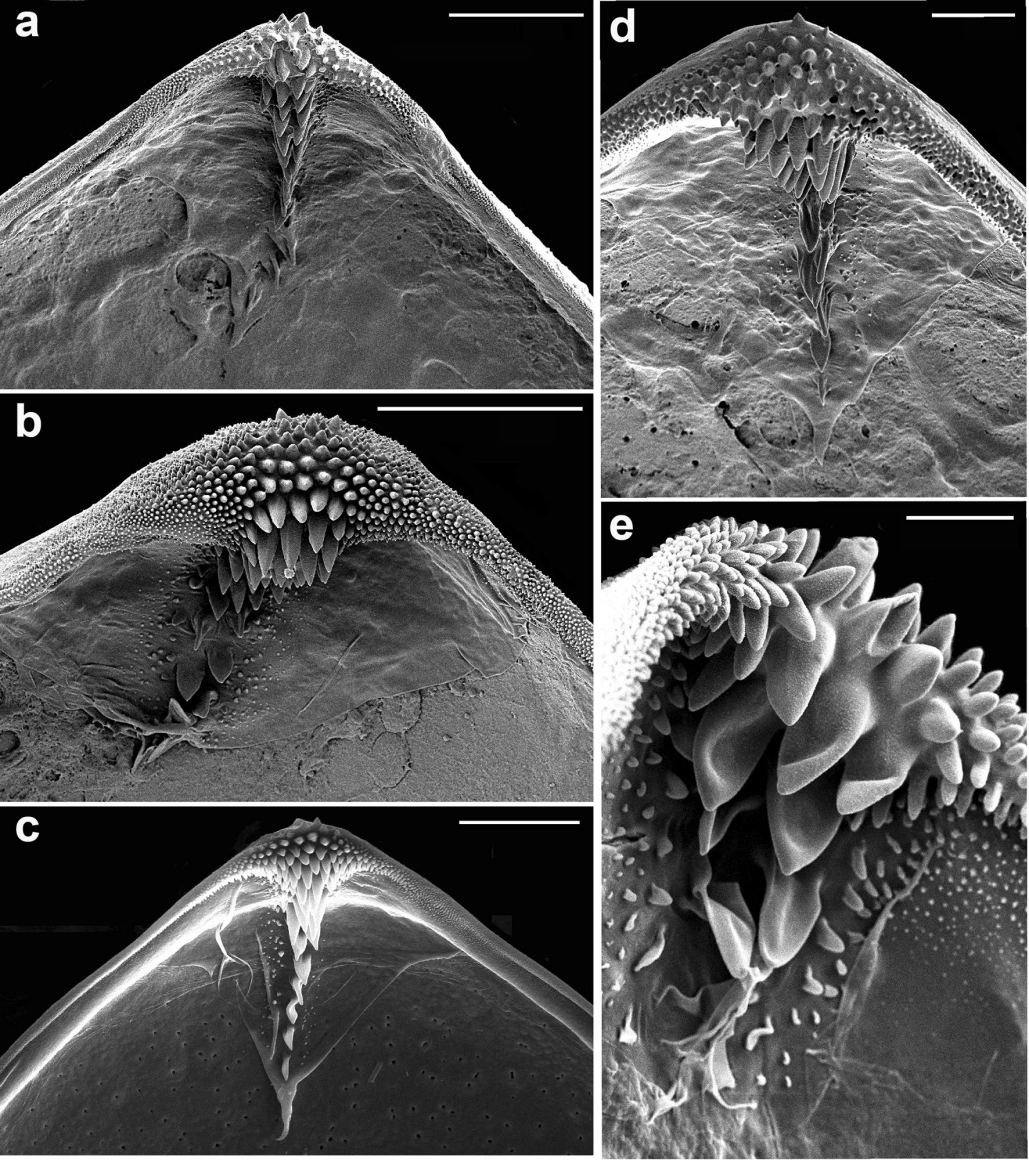
Hooks of *Buldowskia suifunica* mature glochidia (**a**–**d**) and macrospines on a hook (**e**). (**a**, **b**) Solenoye Lake, 22/VI/2017; (**c**) Kamyshevoe Lake, 16/IX/2017; (**d**) Utinoye Lake, 11/XI/2005; (**e**) Solenoye Lake, 22/VI/2017. SEM. Scale bars 50 µm (**a**–**c**), 20 µm (**d**), 10 µm (**e**).

Stylyform hook on the ventral angle of each glochidial valve has a shape of triagular plate (Fig. 2) and is 107–178.5 µm in length, which is more than 30% of the valve height. The hook is covered by lanceolate macrospines (up to 17 µm in height) arranged in 2–3 diagonal rows near the ventral terminus and reduced to a single row distally (Fig. 5a–d). Microspines and micropoints (less than 1 µm in height) cover the entire ventral terminus and less than one-third of the hook lateral lobes, continuing as narrow stripes along the macrospines almost to the end of the hook stylet (Fig. 5e).

**Figure 6:**
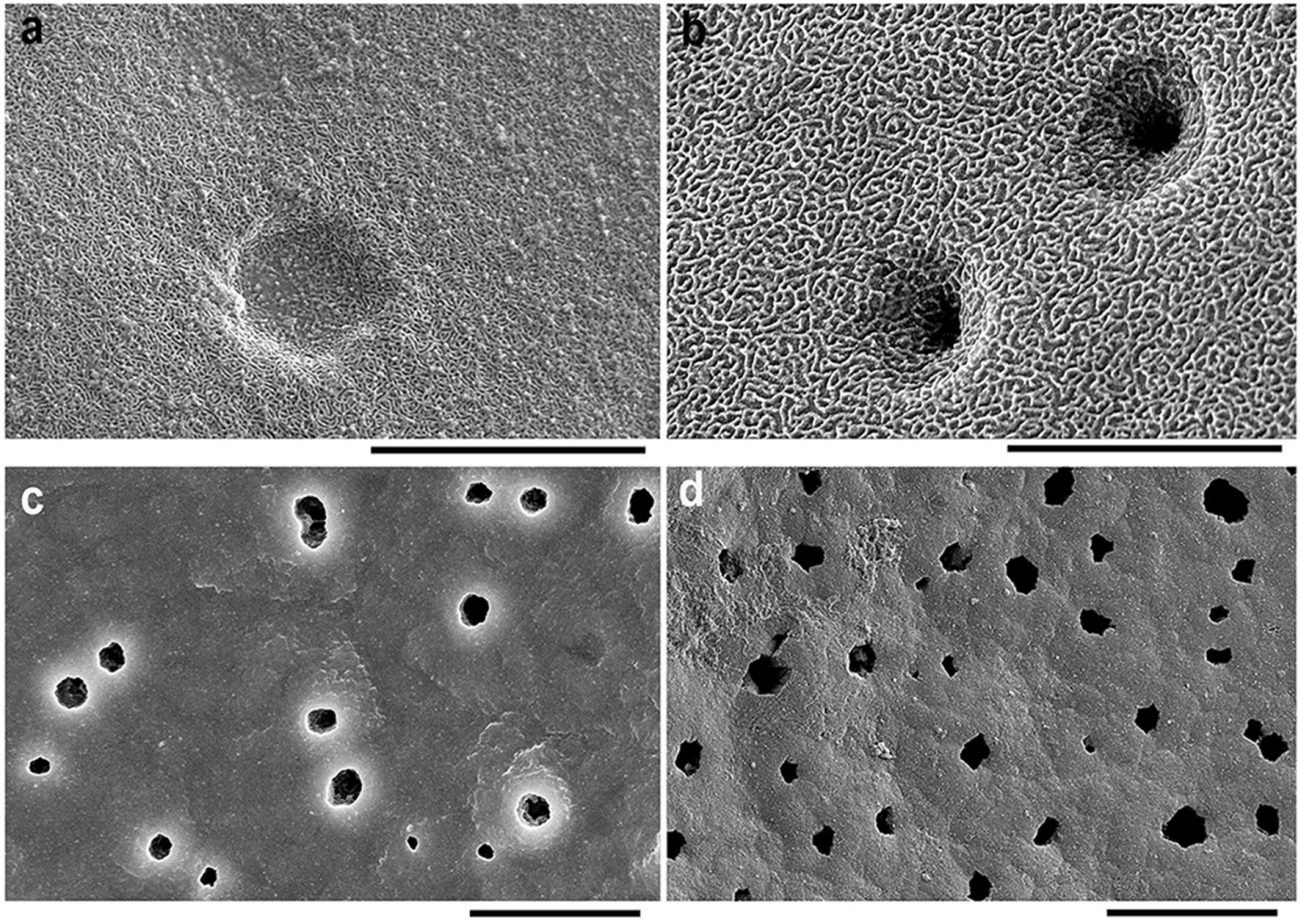
Exterior and interior glochidial valve surfaces. (**a**) Exterior microsculpture in the central part of the valve (Utinoye Lake, 11/XI/2005); (**b**) exterior microsculpture near valve rim (Solenoye Lake, 22/VI/2017); (**c**, **d**) interior pores in central part of valve (Kamyshevoe Lake, 17/IV/2022 and Utinoye Lake, 11/XI/2005). SEM. Scale bars 5 µm (**a**, **b**), 10 µm (**c**, **d**).

The glochidial shell consists of two layers, a thin outer and a thick inner layer. The outer shell layer forms a specific exterior microsculpture, which is loosely looped in *B. suifunica* glochidia, resembling a loose net, wherein the net becomes multilayered in the central part of the glochidial valves, with distinct curved lines going over the main loop pattern (Fig. 6a, b). The inner layer is thick and porous (Fig. 6c, d). The outer ends of the pores are filled with an organic matrix early in the brooding process and later covered by the outer sculpted layer.

The single adductor muscle consists of fibers stretched from one valve to another (Fig. 2d). The larval thread emerges from a canal located at the center of the ventral plate. Together with most anodontins taxa, *Buldowskia* is characterized by a long brooding period because the bivalves of the genus spawn their gametes in the second half of summer, brood the glochidia in marsupia over the winter, and release them from early spring to early summer, depending on water temperature and seasonal fish activities^28^. It can be assumed that by the time of spawning, all glochidia should be fully developed and have hooks; however, glochidia of *B. suifunica*, collected in April and taken from a single gill demibranch, presented the entire range of larval shells, from very immature, with rounded shape and only half the size of mature larva, to mature, round-triangular in shape and with a fully developed hook (Fig. 3). The color of the outer gill demibranches indicates the degree of glochidia maturity. Swollen dark-brown outer demibranches indicates that the majority of *B. suifunica* larvae are already mature. In this study, we registered mature glochidia in late April (before release), late June, September and November (Table 1). Bivalves collected in May and early June had empty gills, which confirms the statement about the brooding of mature larvae in late spring. The following stages of glochidia development were marked in all samples: immature larvae with cap-like shells, immature round glochidia, immature subtriangular glochidia without hooks, and finally, mature glochidia with hooks (Fig. 4). Visible growth lines on the outer surface of the glochidial shells indicate ongoing larval development, whereas less pronounced growth lines or almost invisible growth lines indicate mature glochidial shells (Fig. 4f). First, thin and pale immature shells (Fig. 4a) become thicker and darker (Fig. 4d), and the hooks grow during the last stage of glochidia development (Fig. 4g).

### Acetylated α-tubulin-lir structures in glochidia

**Figure 7:**
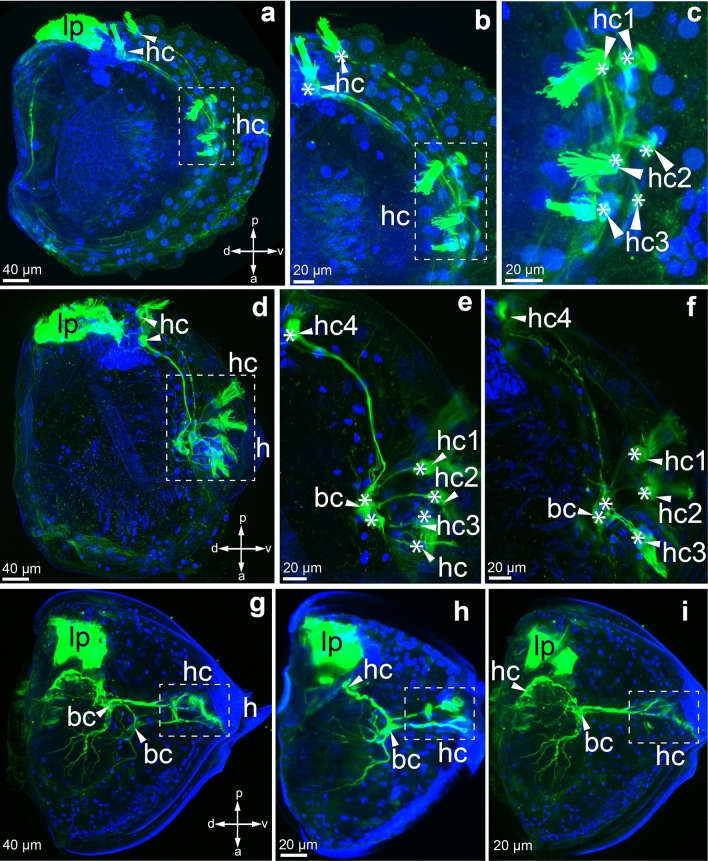
Acetylated alpha-tubulin-immunopositive reaction at the stages of development of *Buldowskia suifunica* glochidia. Green—immunoreaction to acetylated α-tubulin, blue—DAPI. (**a**–**c**) Tubulin-immunopositive reaction in immature larvae; (**d**–**f**) tubulin-immunopositive reaction in intermediate stage larvae; (**g**–**i**) tubulin-immunopositive reaction in mature larvae. Abbreviations: *bc* basal cells, *hc* hair cells, *lp* lateral pit, *h* hook. Asterisks denote the nuclei of cells. Scale bars 40 µm (**a**, **d**, **g**), 20 µm (**b**, **c**, **e**, **f**, **h**, **i**).

Unlike other freshwater mussel studied earlier, *Buldowskia suifunica* contains larvae in the gills that are at different stages of development (Fig. 7). We found small immature larvae still without hooks, glochidia with underdeveloped hooks and large mature larvae with well-developed hooks in the same gill of adult mussel. This fact allowed us to study larvae with different parameters, such as size, shell shape, and presence of hooks.

Antibodies against acetylated α-tubulin were used to visualize the hair cells of *B. suifunica* glochidia (Fig. 7). Immunoreactivity to acetylated α-tubulin revealed that the bodies of the hair cells had multicilia and nonciliated cells with processes (Fig. 7). All the immunodetected α-tubulin structures were bilaterally symmetrical and found on both glochidia valves (Fig. 7).

The presence and structure of the larval sensory system turned out to be an important criterion for determining the maturity of larvae. Indeed, according to the morphology of the sensory system, it was possible to determine what stage of development the larva were in. Based on the complexity of the glochidial sensory system, after analyzing all the larvae taken from the same mussel gill, we were conditionally able to divide the larvae into three stages: immature larvae (1st stage) (Fig. 7a–c), intermediate larvae (2nd stage) (Fig. 7a–c) and mature glochidia (3rd stage) (Fig. 7g–i). In immature glochidia, tubulin antibody revealed the bodies of four paired hair cells arranged in a linear manner. Thus, four immunopositive cells (8 cells in total) lie on one tubulin-positive line, where three pairs of sensory hair cells are located near the ventral margins of the larval shells (Fig. 7b, c) and the fourth pair of sensory cells is located more dorsally and close to the tubulin-immunopositive lateral pits (Fig. 7a, b). Tubulin-immunopositive cilia in all bundles of sensory hairs differed in length when carefully examined in the enlarged picture (Fig. 7c). Paired nonhair cells (basal cells), which are in contact with all hair cells and with each other (Figure), appeared in the more developed larva of the intermediate (2nd) stage, differing from the glochidia of the earlier 1st stage (Fig. 7d–f). The results of immunostaining showed that basal cells were star-shaped with a large number of processes (Fig. 7e, f). Mature glochidia can be clearly distinguished by the bright autofluorescent hooks of the larva (Fig. 7g–i).

Immunostaining showed the presence of both hair cells and basal cells in mature larvae (Fig. 7h, i). Unlike the previous stage of development, the basal cells in mature glochidia have new thin processes extending deep into the body of the larva, covering the central muscle adductor (Fig. 7g, i). We assume that basal cells are neurons that collect sensory information from hair cells and transmit it to the muscle to regulate contraction. Thus, in addition to pronounced external morphological properties (size, hook), tubulin immunostaining identifies the degree of development of glochidia and reveals the complex structure of the larval sensory system.

### Muscle system of glochidia

**Figure 8:**
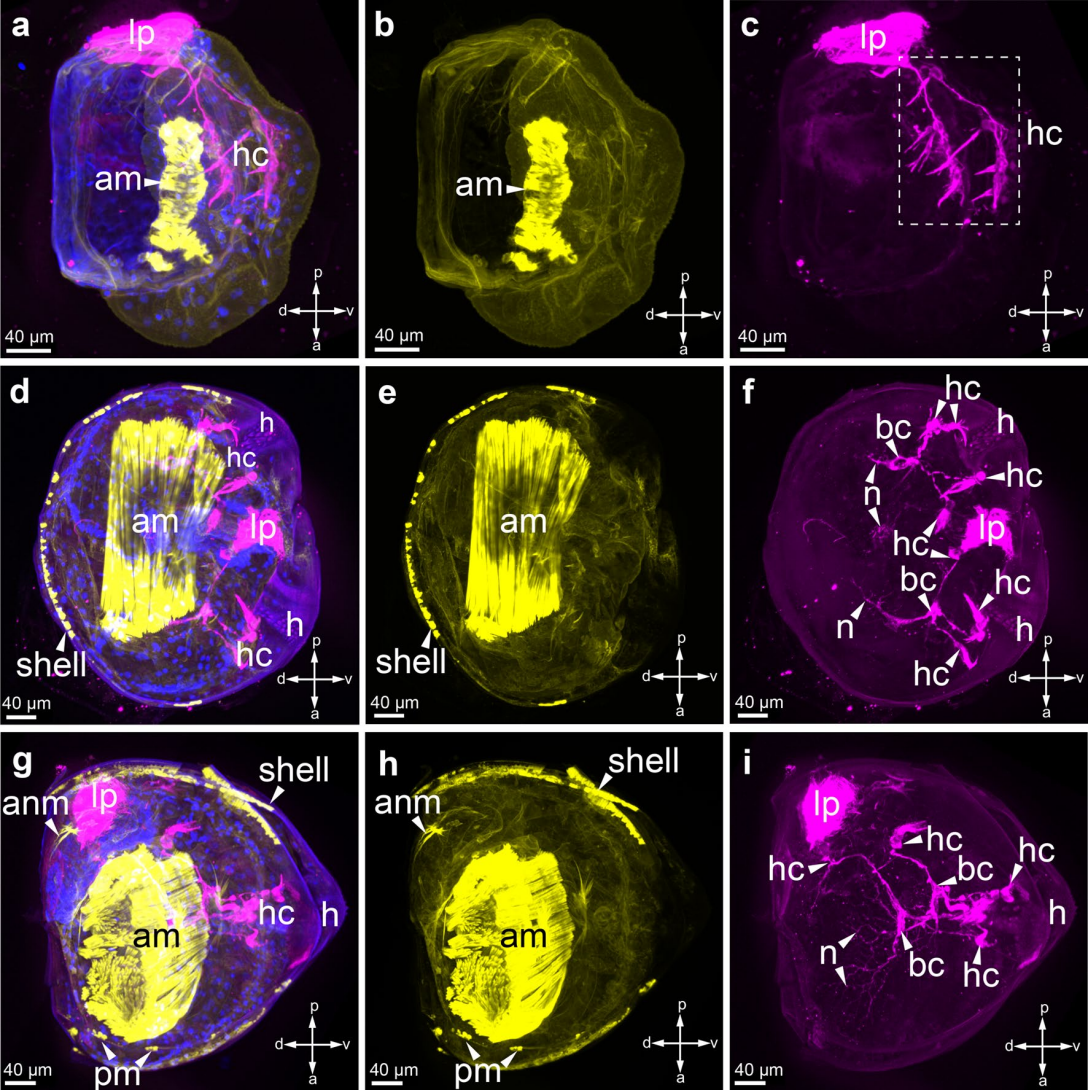
Muscle system of *Buldowskia suifunica* glochidia at different stages of development. Muscles were detected by phalloidin staining (yellow). The muscular structures are represented together with the alpha-tubulin-immunopositive sensory system of larvae (magenta) (**a**, **d**, **g**) and DAPI (blue) or separate muscles (**b**, **e**, **h**) or tubulin (**c**, **f**, **i**) in immature larvae (**a**–**c**), in intermediate stage of larvae (**d**–**f**) and mature glochidia (**g**–**i**). The smooth larval adductor muscle is located in the central part of the glochidia, and larval anterior and posterior muscles are located on the edges of the shell. Muscle bundles appear close to the smooth larval adductor muscle. Abbreviations: *am* adductor muscle; *anm* anterior muscle; *hc* hair cells; *n* neurites; *h* hook; *pm* posterior muscle. Scale bars 40 μm.

In the early stages of larval development, the central adductor muscle is already clearly visible (Fig. 8a, a1). At this stage, there is no contact of tubulin-immunopositive sensory cells with the central adductor (Fig. 8a, a2). At the intermediate (2nd) stage of glochidia development, the size of the adductor muscle is much larger (Fig. 8b, b1). In the open shell, the processes of basal tubulin-immunopositive cells began to surround the muscle (Fig. 8b, b2). In the center of the open glochidium, several cilia of the oral plate can be distinguished (Fig. 8b2). In mature larvae of *Buldowskia suifunica*, in addition to the central adductor, additional muscles appear—these are the anterior and posterior muscles, as well as a separate muscle bundle above the adductor muscle (Fig. 8c, c1). At this stage of larval development, numerous thin processes of basal tubulin-immunopositive cells innervate the central adductor muscle (Fig. 8c, c2). Indeed, basal cells are neuronal cells involved in muscle innervation. Elucidating the transmitting properties of the supposed neurons became our next task.

### FMRFamide-lir larval structure in glochidia

**Figure 9:**
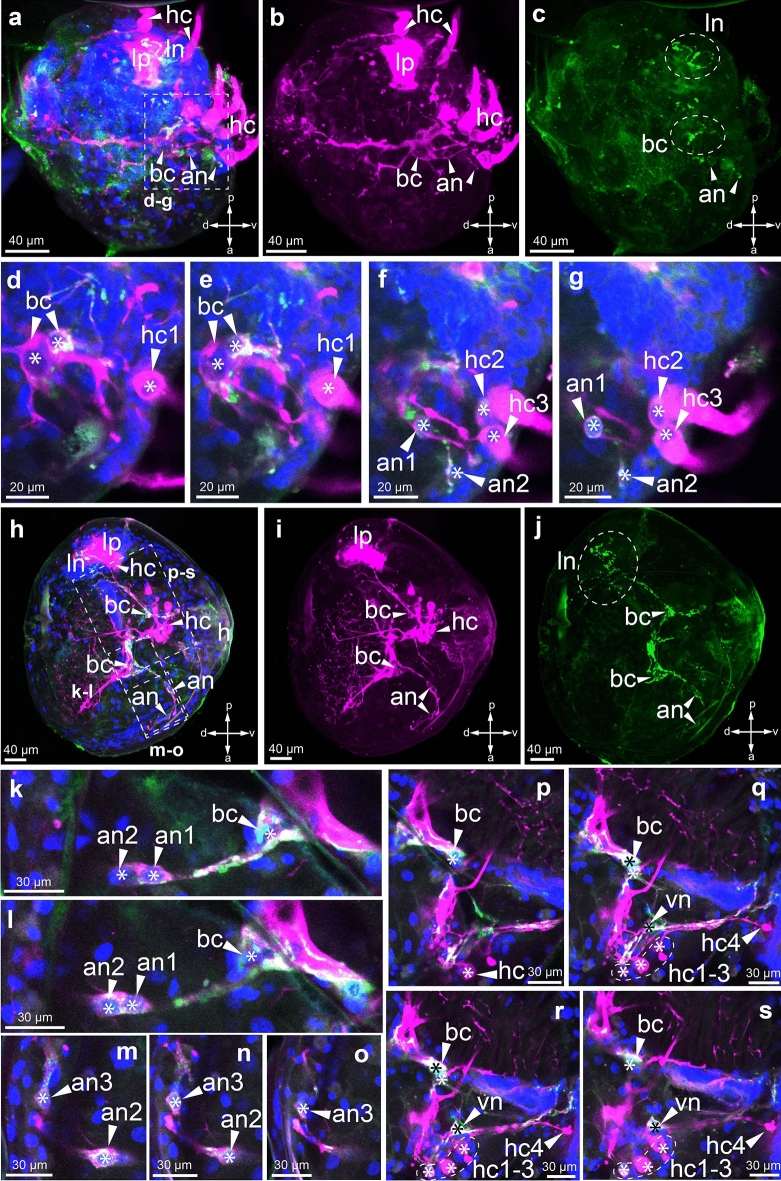
FMRFamide-immunopositive reaction at the stages of development of *Buldowskia suifunica* glochidia. Green—immunoreaction to FMRF, magenta—immunoreaction to tubulin, blue—DAPI. (**a**–**g**) Intermediate stage of larvae; (**h**–**s**) mature larvae; (**a**–**c**) and (**h**–**j**) all Z-projections. In the intermediate stage of larval development, FMRFamide revealed basal neurons, processes and anterior cells (**a**–**c**). FMRFamide-immunopositive neurites radiate from paired basal cells to the central ciliated cells (**d**–**f**) and short process of basal cells connecting to the small FMRFamide-immunopositive anterior cell (an1) (**e**, **f**). On the opposite side, the basal cell neurites connected with the second FMRFamide-immunopositive anterior cell (an2) (**f**, **g**). In mature glochidia, FMRFamide immunoreactivity revealed neurites associated with basal neurons located near the lateral pit (**h**–**j**). FMRFamide immunoreactivity detected anterior processes of basal cells, which connect to two anterior neurons (**k**, **l**). The anterior cells, in turn, communicate with a third separate elongated anterior neuron (**m**–**o**). Another ventral neuron is found (**p**–**s**), which is connected to the basal cells through the FMRFamide process (**p**, **q**). Basal neurons are stretched through the central cell by both tubulin processes and FMRFamide neurites to the lateral ciliary cell (**q**, **r**). Abbreviations: *an* anterior neurons, *pk* ciliated cells, *bk* basal cells, *bn* basal neurons, *ln* lateral neurites, *la* lateral fossa. Asterisks denote the nuclei of cells.

At the early stages of *B. suifunica* glochidia development, we did not detect FMRFamide-immunopositive structures (data not shown). At the intermediate stage of glochidial development, antibodies to FMRFamide revealed basal neurons, processes and anterior cells (Fig. 9a–c). Neurites radiate from the smaller paired basal cells to the central ciliated cells (Fig. 9d–f). From the larger basal cell, a short process connects to the first small anterior cell (Fig. 9e, f), and from it on the opposite side is also a process connecting to the second anterior cell lying near the cilia cells (Fig. 9f, g). In addition, FMRFamide immunoreactivity is shown by lateral neurites near the lateral pit (Fig. 9c). This means that the lateral pit is innervated by FMRFamide immunopositive processes extending from the basal neurons.

In mature glochidia, FMRFamide immunoreactivity revealed many novel structures (Fig. 9h–s). Antibodies against FMRFamide revealed a network of lateral neurites associated with basal neurons located near the lateral pit (Fig. 9h–j). In addition to the basal cell bodies (neurons), FMRFamide immunoreactivity detected their anterior processes, through which they connected to two nearby anterior neurons (Fig. 9k, l). The anterior cells, in turn, communicate with a third separate elongated anterior neuron (Fig. 9m–o), from the dorsal end of which short neurites extend (Fig. 9m–o). Next to the ventral ciliated cells, another ventral neuron is found (Fig. 9p–s), which is connected to the basal cells through the FMRFamide process (Fig. 9p, q). Basal neurons are stretched through the central cell by both tubulin processes and FMRFamide neurites to the lateral ciliary cell (Fig. 9q, r).

### 5-HT-lir larval structures in glochidia

**Figure 10:**
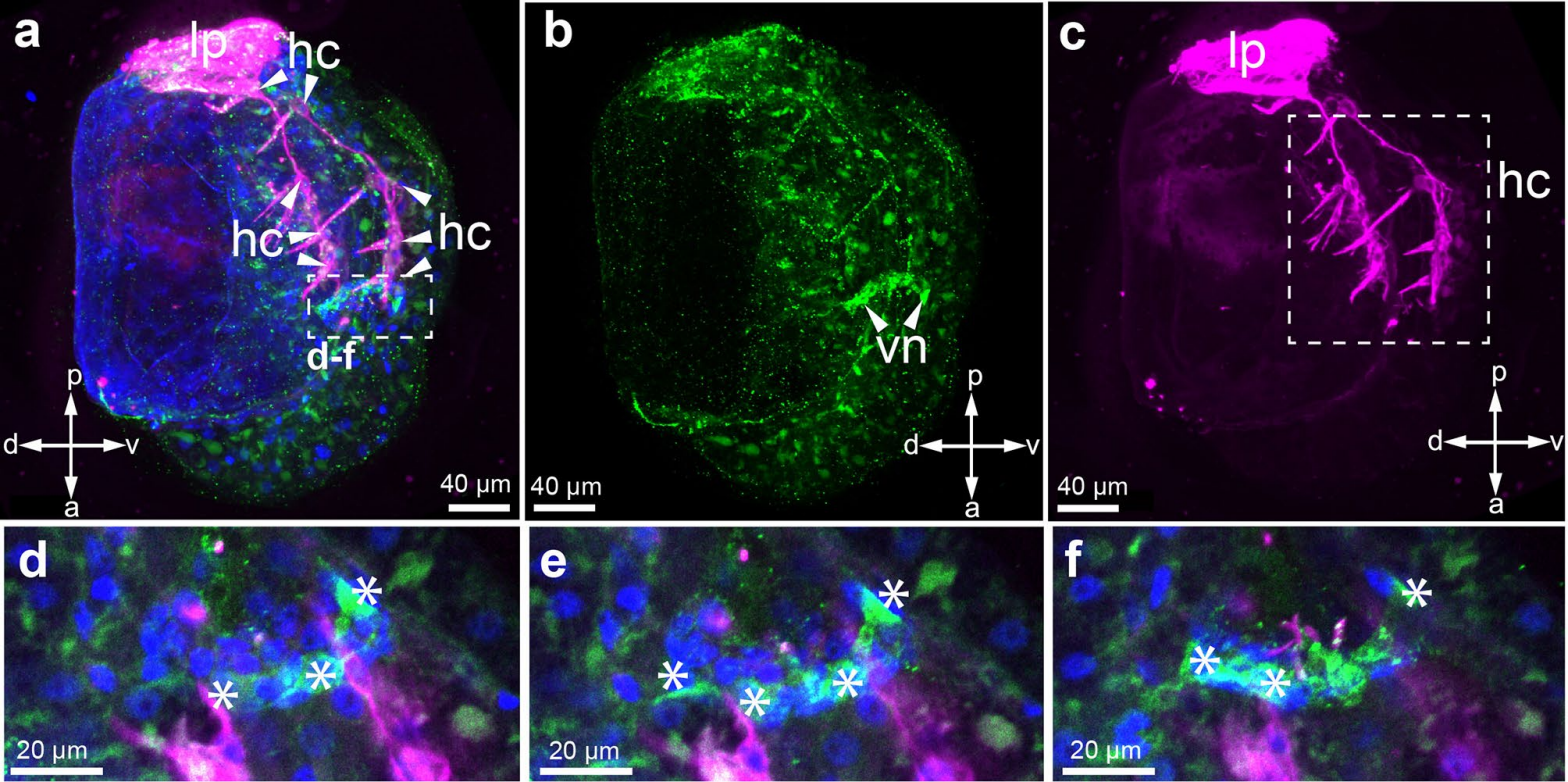
5-HT-immunopositive reaction in the immature larvae of *Buldowskia suifunica* glochidia, whole mount (**a**-**c**), and magnification of 5-HT-immunopositive neurons (**d**-**f**). Green—immunoreaction to 5-HT, magenta—immunoreaction to tubulin, blue—DAPI. Abbreviations: *hc* hair cells, *lp* lateral pit, *vn* ventral neurons. Scale bars: 40 μm (**a**-**c**), 20 μm (**d**-**f**).

Serotonin immunoreactivity was detected in *B. suifunica* at the early stages of glochidia development (Fig. 10). No further developmental stages with serotonin-containing structures were detected. In the ventral part of the glochidium, between tubulin processes of hair cells, paired serotonin-immunoreactive flask-shaped cells without cilia were detected (Fig. 10a–c). These structures consist of two pairs of central neurons: large teardrop-shaped and smaller cone-shaped neurons (Fig. 10d, e). No processes or neurites associated with these neurons were observed.

## Discussion

Studies on the structure of glochidia using light microscopy are very common^18,27^. With the help of scanning electron microscopy, new tiny details of the structure of glochidia were obtained^15^, and these data have become useful for the interpretation of phylogenetic relationships between freshwater mussel taxa^13,14,17^. Since shell sizes generally do not have interspecific variability, increasing data may lead to the proposal of unionid taxa differentiation according to the microsculpture of the outer surface of their shells^13,16,17^. There were no such data on glochidia development and the difference in this process between unionid taxa before our study.

Glochidia development from unfertilized eggs to mature snapping larvae with protruding larval threads and formed hooks includes the following intermediate stages: 4-cell stage, morula with two macromeres, blastula/gastrula stages, trochophore-like stage, early shell formation («patch» stage), late shell formation («bearded» stage), and closed glochidium («D» stage)^45^. Secretion of the shell gland cells in the dorsally located ectodermal region leads to the formation of a thin, cap-like shell^18^. Later, the shell gradually develops, becomes thicker and bivalve. The simultaneous presence of mature and developing immature larvae in a single gill demibranch was noted for all investigated samples of *Buldowskia* glochidia from various water basins^14^. Indeed, in a certain period of glochidia development in unionids, some minor part of larvae in a single gill demibranch may differ by size and shape from most glochidia because of ongoing development; however, eventually, all larvae in the gill demibranch will be mature. For *Buldowskia*, even though it would be logical to see only mature glochidia, we found larvae at different stages of development, and the reason for such asynchrony is still unknown. Previously, it was shown that long-term breeders have very loosely packed glochidia within the marsupia, which allows them to be released in small numbers^46,47^. Such a strategy improves the survival of glochidia. As long-term breeders, *Buldowskia* mussels release some of their mature glochidia when they detect the presences of fish hosts, so the asynchrony of larval development may increase the final chances of infestation. When comparing the completely developed mature glochidia, there was no clear heterogeneity of shell sizes between various samples and populations of *Buldowskia*^14,30^.

Hair cells are mechanoreceptor-type cells with a bundle of cilia that respond to mechanical action. In invertebrates, whose cells are also often multiciliary in nature and presumably act as chemosensory and/or mechanosensory organs in Lophotrochozoa, mechanoreceptors are comparable to the apical organ^48^. Hair cells can act as primary sensory cells, which are specialized neurons with axons capable of perceiving stimuli and generating a nerve impulse, and secondary sensory cells that form synapses with sensory neurons transmitting impulses.

Larvae of marine mollusks (trochophores and veligers) have a multiciliary apical organ^49,50^, and larvae of freshwater mussel (glochidia) have sensory ciliated (hair) cells that develop from specialized ectodermal cells and contain several cilia in the larval mantle. The number and location of bundles of sensory hairs in the mantle of glochidia may be different; therefore, this feature may be suitable for the taxonomy of mollusks. For example, the bivalve mollusk *Anodonta arcaeformis* (Heude, 1877) (Unionidae, Anodontinae) has two types of ciliated cells in the larval mantle^33^. Interestingly, the cells of the first type have a bundle of protruding cilia, that presumably perceive chemical stimuli, in three isolated and highly specialized mantle cells^18^. The function of another type of cilia located on the posterior edge near the lateral pits remains unknown. Only two pairs of bundles of sensory hairs were identified in *Margaritifera margaritifera* (Linnaeus, 1758) and *M. auricularia* (Spengler, 1793) (Unionoidea, Margaritiferidae), rather than the four ones registered in the glochidial softbody of most other Unionoidea^6,51,52^.

**Figure 11:**
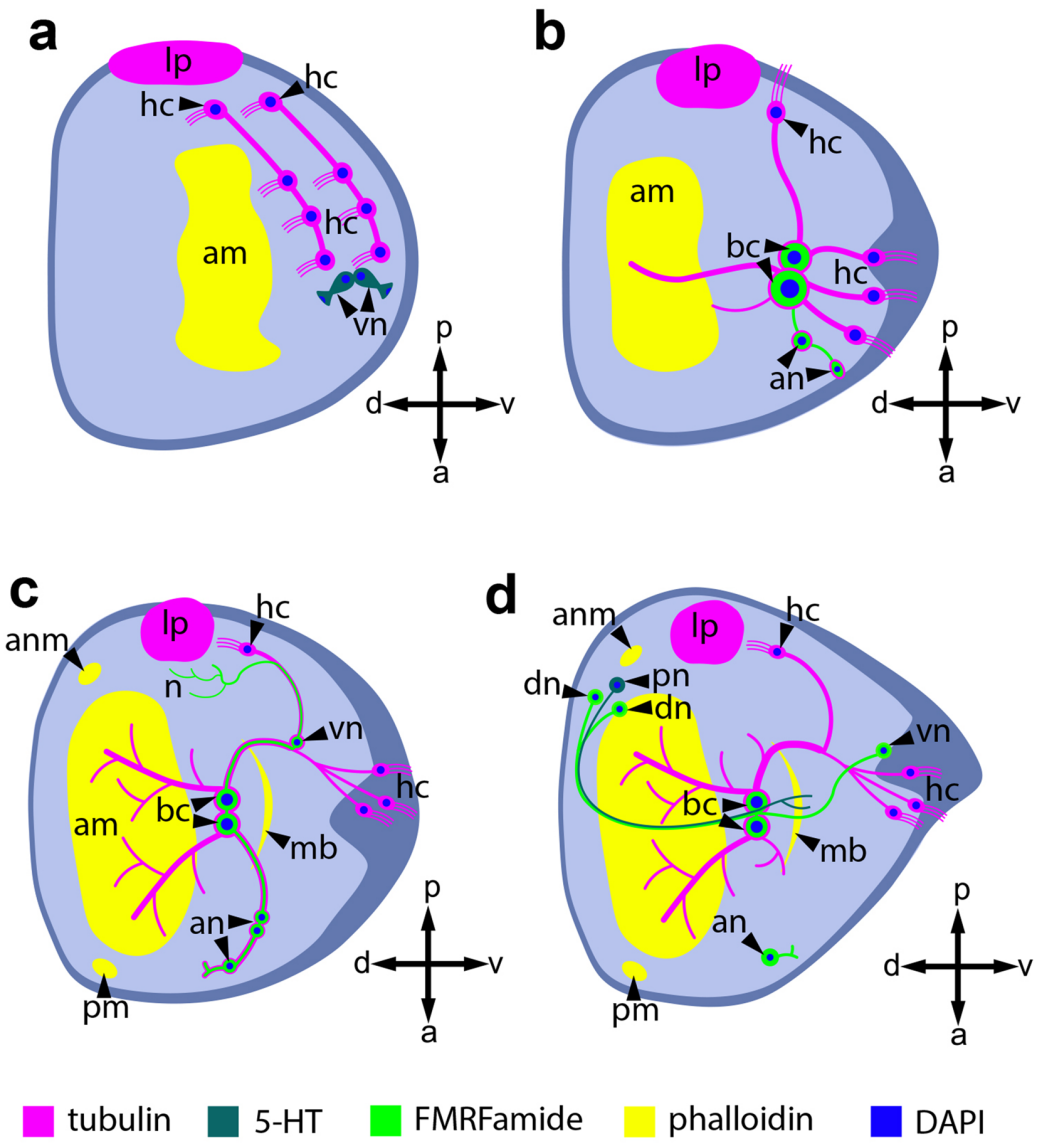
Schematic representation of the muscle, sensory, and nervous system of *B. suifunica* glochidium at various stages of development (**a**, **b**, **c**) and *N. douglasiae* mature glochidium (**d**). (**a**) Immature larvae, (**b**) intermediate stage, (**c**) mature glochidium, (**d**) mature glochidium of *N. douglasiae*. Axes: *a* anterior, *p* posterior, *d* dorsal, *v* ventral. Color keys: magenta—tubulin; dark turquoise—5-HT; green—FMRFamide; yellow— muscles. Abbreviations: *am* adductor muscle; *an* anterior neuron; *anm* anterior muscle; *bc* basal cell; *hc* hair cell; *lp* lateral pit; *mb* muscle band; *n* neuritis; *pm* posterior muscle; vn ventral neuron.

Earlier, we showed that *Nodularia douglasiae* glochidium has four pairs of bundles of ciliated cells, wherein each glochidial valve has three bundles of sensory hairs densely located on the ventral edge near hooks and one bundle located more dorsally near the lateral pit^40^. We found very similar localization in *Buldowskia suifunica* glochidia, with three pairs of bundles of sensory hairs on the ventral edges of glochidial valves and one pair of bundles located more dorsally (Fig. 11a). These data are consistent with previously obtained morphological data on the structure and location of sensory hairs in *Buldowskia* glochidia from Korea, where each glochidial valve had three bundles of sensory hairs located close to the basal part of the hook and one bundle indicated near the adductor muscle at the larval thread canal^33,35,53^. It should be noted that the bundles of sensory hairs in the larvae of both species, *N. douglasiae* and *B. suifunica*, are connected with nonciliated cells (basal neurons), which have tubulin/FMRFamide immunoreactivity, that interact with other neurons through neurites (Fig. 11b, c). We have not detected an apical organ during the development of *B. suifunica* glochidia, even though this structure is present in most molluscan and other lophotrochozoan larvae^42,44,49,50^. The free-swimming larval stage of parasitic flatworms from Trematoda, miracidium larva, also does not have an apical organ^54,55^. It is likely that both types of larvae, glochidium and miracidium, are committed to finding a host in the aquatic environment, and the absence of an apical organ can be explained by a parasitic lifestyle at subsequent stages of development.

The structure of the nervous system of *N. douglasiae* glochidia was described using 5-HT, FMRFamide, and tubulin immunostaining^40^. A comparative analysis showed that the nervous systems of *B. suifunica* and *N. douglasiae* glochidia are arranged differently. First, FMRFamide dorsal neurons are present in *N. douglasiae* glochidia but are absent in *B. suifunica* larvae at all developmental stages. Second, in *N. douglasiae* glochidia, double immunopositivity for a-acetylated tubulin and FMRFamide is present only in basal cells, but in *B. suifunica* glochidia, it is also detected in anterior neurons and in processes of lateral ciliated cells in the last stages of larval development. We did not find neuronal clusters of cells forming ganglia during any studied developmental stages of *B. suifunica* glochidia, although some researchers^52^ claimed that glochidia of *Anodonta anatina* (Linnaeus, 1758) and *Pseudanodonta complanata* (Rossmaessler, 1835) had three pairs of rudimentary ganglia (cerebral, pedal and visceral). We assume that rudimentary ganglia in *B. suifunica* glochidia are formed during or after infection of the host.

The serotonin nervous system of glochidia is not as morphologically diverse as FMRFamide. Unlike the glochidia of *N. douglasiae*^40^, the glochidia of *B. suifunica* have serotonin-containing ventral cells only at the early stages of development; at subsequent stages, serotonin-containing elements cannot be detected (Fig. 11).

Although smooth adductor muscle appears from mesoblast cells in the early stages of glochidial development^55^, before the appearance of the shell, it is not functional. After the formation of the bivalve shell, a well-developed adductor muscle is capable of rapid contraction and relaxation and, thus, is able to regulate the opening and closing of the shell^18^. In immature glochidia of *B. suifunica,* the unitary central adductor muscle can already be detected by fluorescently labeled phalloidin. It is also noticeable that in the subsequent stages of development, this smooth muscle becomes more massive, and additional muscle bundles appear, for instance, the central one, which was not detected earlier. When studying the morphology of glochidia, in which only one adductor muscle for contraction is developed, the following question arises: when and how are the anterior and posterior adductor muscles of an adult freshwater mussel formed? The histochemical and actin staining of glochidia of the North American freshwater mussel *Utterbackia imbecillis* (Say, 1829) showed complete degradation of the larval adductor muscle during the first few days of metamorphosis^39^. A similar situation can be found in marine mollusks, when at the stage of pediveliger, a resorption of striated muscle retractors regulating the movement of velum, which are also resorbed, occurs^42^. The molecular and cellular mechanisms of organ change and transformation during metamorphosis in freshwater mussel will be the subject of further research.

## Conclusions

In this work, we confirmed some glochidial features distinguishing the freshwater bivalve *Buldowskia suifunica* from all other Far Eastern anodontins. First, glochidia development is asynchronous given that larvae taken from the same gill at the same time were in different stages of development. Then, the outer layer of *B. suifunica* glochidial shells were loosely looped, resembling a loose multilayered net, with distinct curved lines going over the main loop pattern. To our knowledge, this is the first immunocytochemical study of the sensory and nervous systems of *B. suifunica* glochidia. We detected the presence of serotonin- and FMRFamide-ergic elements of the nervous system, as well as tubulin, for the detection of cilia cells in parasitic larvae of freshwater bivalves at three stages of larval development (immature, intermediate stage and mature larvae). The alpha-acetylated tubulin-immunopositive sensory system consists of four pairs of multiciliated hair cells as well as tubulin-immupositive nonhair cells in three stages of larval development (immature, intermediate and mature glochidia). FMRFamide and tubulin expression was found in all neurons. FMRFamide has been detected in basal cells (neurons), their neurites and anterior neurons and has been shown to participate in innervation of the lateral pit but not of the adductor muscle. For the 5-HT nervous system, only four 5-HT-lir neurons were detected in *B. suifunica* glochidia. The glochidia muscle system includes a unitary central massive adductor muscle innervated by tubulin-immunopositive processes and some minor muscle bundles. Based on previously data of freshwater unionin mussels glochidia *Nodularia douglasiae* (Griffith & Pigeon, 1833) and presented here results on *Buldowskia suifunica* (Lindholm, 1925) we may conclude that some glochidial nervous, sensory and muscle elements differ from marine bivalve veliger larvae due to the location and composition of FMRFamide and 5-HT cells and the absence of apical organs^40^. Further studies of the role of neurotransmitters in the behavior and the parasite-host relationship of glochidia, and the processes of reorganization of the larval nervous system into that of adult mussel are interesting and relevant areas of research for unionids in the future.

## Materials and methods

### Specimen sampling

Specimens of *Buldowskia suifunica* were collected from three localities in Primorye, the Russian Far East. The collections of dry shells as well as samples of gills with glochidia preserved in 75% ethanol were stored at the Laboratory of Freshwater Hydrobiology, Federal Scientific Center of the East Asia Terrestrial Biodiversity, Far Eastern Branch of the Russian Academy of Sciences, Vladivostok (FSCEATB FEB RAS).

After removing the soft parts, the shells of adult mussels were washed, dried and labeled in the laboratory, and photographed using a digital camera. Identification was made according to the most recent taxonomic revisions^23,24^ and confirmed by DNA data of mollusks collected in the abovementioned localities^23^.

### Procedures for light and scanning electron microscopy

Samples with glochidia initially fixed in 75% alcohol were used to investigate the morphology of glochidial shells. To prepare larval shells for light and scanning electron microscopy, standard procedures were used^14,41^. To prevent any deformation or destruction, only chemical cleaning was used; first, the glochidia were washed several times in distilled water and cleaned in a 5% KOH solution for 1.5–2 h; after that, the cleaned shells were again washed several times in distilled water and dehydrated in an alcohol series (80, 90, and 96%); finally, the glochidial shells were mounted on permanent slides for light microscopy as well as on stubs for scanning electron microscopy and photography. To check the soft parts of glochidia by scanning electron microscopy, some larvae underwent a shortened cleaning procedure in which only the outside of the shells were cleaned in alkali, preserving the inside soft tissues as best as possible. For scanning electron microscopy, sputter coating with chromium or gold was used immediately after drying the samples on a stub to exclude the possibility of deformation of the hooks and outer shell layer.

Larval shells were measured using a Nikon Alphaphot-2 YS2 light microscope and a Zeiss MERLIN scanning electron microscope. The following larval shell measurements are used in this work: length and height of the shell, length of the hook, and length of the ligament^14^.

Photographs of glochidia were obtained on a Zeiss MERLIN scanning electron microscope at the Biology and Genetic Engineering Center for Collective Use of the FSCEATB FEB RAS.

### Immunostaining procedure

Glochidia-filled gills from adult mussel were fixed in a 4% PFA solution with phosphate buffer (PBS, 100 mM Na3PO4, 140 mM NaCl, pH 7.4) for 2–3 h at room temperature^42^. Fixed larvae were isolated from the gills and washed in 0.1 M PBS. The samples were dehydrated using methanol solutions with increasing concentrations (25, 50, 75, and 100% methanol) and stored in 100% methanol at -20 °C. Immediately before immunostaining, the larvae were transferred from 100% methanol to PBS by changing them to solutions with a decreasing concentration of methanol. Samples were incubated for 1 h in 5% ethylenediaminetetraacetic acid (EDTA) in PBS at room temperature for shell decalcification, which is necessary to reduce the adhesion of antibodies to the shell and improve the quality of immunostaining^42–44^. Samples were washed in PBS with the addition of 0.1% Triton X-100 (PBST) for 3 × 30 min. Then, the samples were incubated overnight in a blocking solution (10% donkey normal serum, 1% bovine serum albumin, 1% Triton X-100, 0.003% NaN_3_ in 0.1 M PBS) at + 4 °C.

The muscular system of *B. suifunica* glochidia was identified using phalloidin conjugated with fluorochrome. To detect nervous structures, 50–80 larvae of each stage of development (early, middle and late) were incubated with primary antibodies (rabbit anti-serotonin (#20080) and anti-FMRFamide polyclonal antibodies ImmunoStar (#20091); goat anti-serotonin polyclonal antibodies, ImmunoStar (20079) at a dilution of 1:1000 together with mouse monoclonal antibodies against acetylated α-tubulin (Santa Cruz, 23950) in a blocking solution for 3 days at a temperature of + 4 °C. Then, after washing in PBS (5 × 10 min), the samples were incubated overnight at 4 °C in a blocking solution with a dilution of 1:1000 and 0.1 μg/mL DAPI with the following secondary antibodies: Alexa Fluor 488 donkey anti-goat IgG (DAG) (Invitrogen, A32814), Alexa Fluor 555 donkey anti-rabbit IgG (DAR) (Invitrogen, A32794), Alexa Fluor 555 donkey anti-goat IgG (DAG) (Invitrogen, A32816), Alexa Fluor 488 donkey anti-rabbit IgG (DAR) (Invitrogen, A32814), and Alexa Fluor 647 donkey anti-mouse IgG (DAM) (Invitrogen, A32787). Finally, the larvae were washed in PBST (5 × 20 min).

### Confocal microscopy

All samples prepared for confocal microscopy were placed on slides in a drop of 70% glycerin. About 50–80 larvae samples of each stage of development stained immunocytochemically were scanned using a confocal microscope LSM 780 (Zeiss, Germany) and Zen software using lasers with the following wavelengths: 405, 488, 555 and 647 nm. All images of larvae were obtained in the Z-stack mode with an optical slice thickness of 1 micron along the Z-axis. The resulting images were converted into projections in the maximum intensity mode. All images were analyzed using ImageJ software (USA).

## Data Availability

The datasets of confocal scans and data from an electronic scanning microscope during the current study available from the corresponding author on reasonable request.
